# Mucosal Vaccination Strategies against *Clostridioides difficile* Infection

**DOI:** 10.3390/vaccines11050887

**Published:** 2023-04-23

**Authors:** Joshua Heuler, Harish Chandra, Xingmin Sun

**Affiliations:** 1 Department of Molecular Medicine, Morsani College of Medicine, University of South Florida, Tampa, FL 33612, USA; 2 Department of Molecular Genetics, Biochemistry and Microbiology, University of Cincinnati College of Medicine, Cincinnati, OH 45267, USA

**Keywords:** *Clostridioides difficile*, mucosal vaccine, passive vaccination, active vaccination, surface protein, toxin, spore protein

## Abstract

*Clostridioides difficile* infection (CDI) presents a major public health threat by causing frequently recurrent, life-threatening cases of diarrhea and intestinal inflammation. The ability of *C. difficile* to express antibiotic resistance and to form long-lasting spores makes the pathogen particularly challenging to eradicate from healthcare settings, raising the need for preventative measures to curb the spread of CDI. Since *C. difficile* utilizes the fecal–oral route of transmission, a mucosal vaccine could be a particularly promising strategy by generating strong IgA and IgG responses that prevent colonization and disease. This mini-review summarizes the progress toward mucosal vaccines against *C. difficile* toxins, cell–surface components, and spore proteins. By assessing the strengths and weaknesses of particular antigens, as well as methods for delivering these antigens to mucosal sites, we hope to guide future research toward an effective mucosal vaccine against CDI.

## 1. Introduction

*Clostridioides difficile* is a Gram-positive, spore-forming anaerobic bacterium [1] that causes most cases of hospital-acquired diarrhea [2]. *C. difficile* infection (CDI) usually arises following antibiotic usage that drives gut microbiota dysbiosis, allowing *C. difficile* to overgrow [3]. Asymptomatic carriers, animal reservoirs, and contaminated food can cause disease outside healthcare settings [4,5,6,7]. Antibiotics, such as vancomycin and fidaxomicin, are typical CDI treatments [8], but antibiotics are ill-suited to clearing CDI permanently because they disturb commensal bacteria, leading to frequent disease recurrence [9]. Antibiotic resistance poses a significant threat to CDI patients [10,11], as strains less susceptible to fidaxomicin and/or resistant to vancomycin have recently emerged [12], demonstrating the need for novel treatments and preventative methods to deal with the growing threat of CDI. Although emerging therapeutic strategies against CDI, such as fecal microbiota transfer (FMT) [13] and phage therapy [14], could help patients already diagnosed with *C. difficile*, a *C. difficile* vaccine could offer a significant economic advantage by avoiding the costs of treating CDI in the first place [15]. A recent simulation model demonstrated that a *C. difficile* vaccine could be a cost-effective disease management tool for both primary and recurrent CDI [15]. Although vaccines against *C. difficile* toxin A (TcdA) and toxin B (TcdB) are undergoing clinical trials [16,17], the failure of the Sanofi inactivated toxoid vaccine [18] raises the need for new insights into *C. difficile* vaccination strategies. Current *C. difficile* vaccine candidates in clinical trials are administered via the parenteral route (Supplementary Table S1 [16,17,19,20,21,22,23,24,25,26,27]). Given that *C. difficile* must first colonize the gut mucosa to cause disease [28], a mucosal vaccine could offer superior protection against CDI by triggering strong mucosal and systemic immune responses. In this mini-review, we summarize the progress made toward developing mucosal vaccines against CDI. We assess the benefits and drawbacks of current mucosal vaccine designs and identify potential candidates for further study.

## 2. Animal Models Used for *C. difficile* Vaccination Studies

Since animal models are critical for evaluating vaccination and host immune responses to CDI, we will briefly summarize the current major animal models of CDI. Compared to clinical studies, animal models provide the advantages of greater subject availability, similar disease severity to human infections, and opportunities to perform highly invasive experiments and tissue sampling [29]. Hamsters have historically been the most prevalent CDI model system. However, numerous other animals have been used, including mice, rats, rabbits, hares, guinea pigs, prairie dogs, quails, foals, piglets, monkeys, and zebrafish embryos [29]. Regarding *C. difficile* vaccines in particular, hamsters are still one of the main model systems used to evaluate vaccine effectiveness, e.g., [30,31,32,33,34,35,36]. As summarized in a prior review [29], hamsters accurately model many aspects of human *C. difficile* infections. As in human CDI cases, antibiotic administration drives hamster gut dysbiosis, allowing for colonization with administered *C. difficile*. Hamsters also display many of the same signs of gut inflammation as humans. On the other hand, hamsters differ from humans when it comes to mortality, as hamsters succumb to CDI in a matter of days and do not typically exhibit diarrhea as humans would. When considering experimental design flexibility, hamsters are also limited by the availability of reagents for immunological studies.

In addition to hamsters, mice have also been frequently used in developing mucosal vaccines against *C. difficile*, e.g., [37,38,39,40], as *C. difficile* toxins drive similar tissue damage and inflammation as is seen in humans with CDI [41]. Like hamsters, mice are typically resistant to *C. difficile* colonization unless given antibiotic treatment beforehand to disrupt their microbiome [42], similar to the typical progression of human CDI. For example, mice treated with cefoperazone, a cephalosporin antibiotic, become susceptible to *C. difficile* infection and serve as a useful model system [43]. This model aligns with prior findings that cephalosporin use in humans is associated with greater CDI risk [44].

One of the primary advantages of mice over hamsters is that mice are less susceptible to death from CDI and thus better facilitate the study of mild, severe, and recurrent CDI [45]. Depending upon the *C. difficile* strain used, the microbiome of the mouse strain being used, and the age of the mice, mice can exhibit different levels of susceptibility to CDI [29,41,46]. On the one hand, *C. difficile* can colonize mice asymptomatically while the mouse continues to shed spores [42], which could be helpful for studying the spread of CDI by asymptomatic carriers. On the other end of the spectrum, recurrent episodes of CDI can also be examined using mouse models with both wild-type [47] and humanized [48] microbiomes.

## 3. Immune Responses to Mucosal Vaccination

Mucosal vaccines could prevent infection by triggering antibody responses through the adaptive immune system [49,50]. Tissue-resident dendritic cells (DCs) and macrophages are the primary cells that directly process antigens in mucosal tissues [50]. Tissue-resident DCs begin as immature, immunoregulatory cells that can mature into migratory, pro-inflammatory DCs that present antigens to T cells within the lymph nodes [51,52]. Antigens can also be transferred to migratory DCs from macrophages and other DCs [51,53]. Meanwhile, gut mucosal macrophages do not migrate to secondary lymphoid organs and thus do not play a role in antigen presentation to T cells in the lymph nodes [53,54]. Even so, there are a number of T cells available in the lamina propria for stimulation by resident macrophages and DCs [52]. T cells, in turn, can stimulate IgA production by mucosal B cells [55].

The gut epithelium layer also contains specialized areas for immune activation known as gut-associated lymphoid tissue (GALT), which samples and processes antigens to induce adaptive immune responses [56]. GALT is further broken down into Peyer’s patches (PPs) in the small intestine and isolated lymphoid follicles (ILFs) in the large and small intestines. Both structures contain M cells [56], which sample antigens from the gut lumen for processing by immune cells [57]. M cells can also transfer vaccine antigens to stimulate adaptive immune responses [50,58]. In PPs, DCs are thought to primarily stimulate B cells through a T cell-dependent mechanism [56,59], whereas DCs in ILFs can perform this role independently of T cells [56].

**Figure 1:**
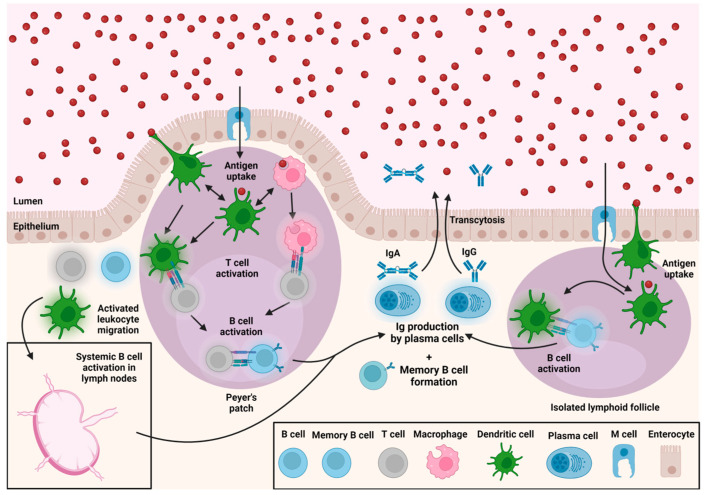
Antibody response to mucosal antigens. Antigens (shown above as red spheres) are sampled in the GALT by DCs and M cells in Peyer’s patch (left) and ILFs (right). Peyer’s patches heavily use T cells to activate B cells, whereas ILFs do not require T cells. B cells can also be activated in lymph nodes (bottom left) following the migration of activated B cells, T cells, and/or DCs from the GALT. The above diagram is not to scale and does not show all mucosal immune processes. A publication license for BioRender content used in the above figure was obtained on 24 February 2023 (agreement number NO251WZ00A).

One of the most important effector cell types in the mucosal immune response is the plasma cell, a terminally differentiated B cell that produces immunoglobulins (Ig) against invading pathogens [60]. Tissue-resident B cells in the gut primarily produce IgA, which, once secreted through M cells [58] or by transcytosis through epithelial cells [61], plays a multifaceted role in targeting pathogens, protecting commensals, and regulating overall mucosal immunity [52]. Up to 15% of mucosal B cells secrete IgG antibodies, but these are more rapidly degraded in the gut than IgA [62]. Mucosal DCs can directly activate B cells to produce antibodies [52]. Some evidence suggests that M cells can also directly stimulate B cells as well [63], but work is ongoing to fully understand this relationship. Figure 1 describes the general processes by which mucosal antigens trigger antibody responses in the gut.

**Figure 2:**
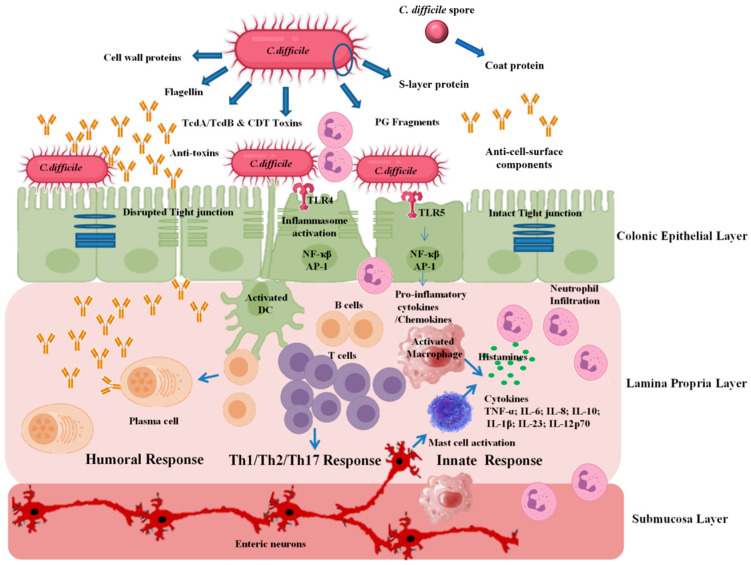
Mucosal immune response against *C. difficile* infection in the gut. In the gut, *C. difficile* spores germinate into vegetative cells, which produce *C. difficile* toxins (TcdA, TcdB, and CDT). Toxins disrupt the tight junctions and epithelial layer and enter into lamina propria and submucosal layers while activating host cells [64]. In response to *C. difficile* surface components, host cells elicit a strong innate immune response via TLR4/5, leading to the activation of inflammasome and induction of pro-inflammatory cytokines and chemokines via nuclear factor κB (NF-κB) and transcription factor AP 1 [65]. Through activation of the inflammasome, mitogen-activated protein kinases (MAPK), and maybe other pathways, toxins can also activate host cells to release inflammatory mediators and recruit neutrophils during inflammatory response [72]. Activation of the antigen presentation by DCs and macrophages stimulate B cells and T cells, which induces a robust humoral antibody response (IgG and IgA) against the major cell surface components and toxins. The T cell response consists of Th1/Th2 and Th17 cell response to neutralize *C. difficile* assault [66]. Activation of the antigen presentation by DCs and macrophages stimulate B cells and T cells, which induce a robust humoral antibody response (IgG and IgA) against the major cell surface components and toxins.

Mucosal immunity against *C. difficile* invasion is highly crucial, as the pathogenesis of this bacteria starts at the gut mucosal interface (Figure 2). *C. difficile* spore germination, adhesion, colonization, and infection in the gut disrupt the intestinal epithelial barrier as a result of its toxin (TcdA and TcdB) secretion, causing the loss of epithelial barrier integrity. Toxin binding through respective receptors on intestinal epithelial cells leads to the disruption of the skeletal structure and the tight junctions, forming a leaky gut [64]. In response to the bacterial invasion and the surface components, host intestinal cells activate the inflammasome, nuclear factor-κB (NF-κB), and activator protein 1 (AP-1), which lead to the secretion of several pro-inflammatory cytokines and chemokines (IL-1a, IL-1β, IL-8, and CXCL1) [65]. This inflammatory response triggers a robust innate immune response. At this point, the antigenic components of the *C. difficile* bacterium also prime the antigen-presenting cells, such as macrophages and dendritic cells (APCs). The APCs further interact with B cells and T cells to trigger humoral and cell-mediated immunity (Th1, Th2, and Th17 responses). The detailed immune response against the *C. difficile* invasion has been extensively reviewed elsewhere [49,50,52,56,62,66].

Briefly, it has been shown that *C. difficile* strains induce Th1/Th2/Th17 and T-reg cell responses. Specifically, hypervirulent *C. difficile* R20291 induces a strong Th1 and Th17 response in terms of IFN-γ+ and IL-17A CD4 T cells compared to the non-virulent *C. difficile* 630 strain in co-cultured murine bone-marrow-derived dendritic cells (BMDCs) and splenocytes [67]. Clinical data with *C. difficile*-infected patients showed a shift from Th1 to Th17 response or Th2 response with the increasing severity of the disease [68]. Further, it is known that, unlike adults, young children show resistance against CDI, and it has been demonstrated that IL-17A produced by γδ T cells are involved in the resistance [69]. Elevated levels of IL-17A and T cell receptor γ expressions have been detected in stool samples of children. In neonatal mice, which are also resistant to CDI, RORγt+ γδ T cells produced significant levels of IL-17. Meanwhile, the protective effect was lost when these IL-17-producing T cells were depleted [69]. 

*C. difficile*-mediated pseudomembranous colitis is an inflammatory disease associated with the dysregulation of immune homeostasis. Mouse models of inflammatory diseases suggest the important role of regulatory T-cells (Treg) in ensuring proper immune system function [70]. Upon encountering an antigen in the gut-associated lymphoid tissues, the naïve CD4+ T cells differentiate to peripheral Treg cells and provide tolerance by maintaining intestinal equilibrium by secretion of the anti-inflammatory IL-10 cytokine. In fact, deletion of IL-10 in mice develops severe colitis [71]. A detailed review of the activation of T cell response has been presented elsewhere in our previous efforts [66]. Therefore, it can be argued that the development of a mucosal vaccine has the potential to give higher protection against *C. difficile* infection.

## 4. Mucosal Vaccination against *C. difficile* Toxins

### 4.1. Passive Mucosal Vaccination against C. difficile Toxins

The *C. difficile* pathogenicity locus (PaLoc) codes for toxin A (TcdA) and toxin B (TcdB), the primary drivers of CDI symptoms [73]. TcdB is thought to be more heavily involved in CDI pathogenesis than TcdA [74], but both toxins cause intestinal damage [75]. TcdA and TcdB bind cell receptors on the colonic epithelia, become endocytosed by the target cells, and glucosylate host GTPases [64]. This glycosylation disrupts cytoskeleton organization, which damages the intestinal epithelium [64].

One method of mitigating the effects of *C. difficile* toxins is passive vaccination, whereby neutralizing antibodies are delivered directly into the body. The advantages of passive vaccination include the high specificity and low toxicity of purified antibodies [76]. Regarding *C. difficile*, multiple studies have utilized animal-derived antibody preparations as passive mucosal vaccines [31,77,78,79,80]. By using polyclonal antibodies, these studies avoided the challenge of determining target epitopes, which continues to be challenging for monoclonal antibody development against TcdB and TcdA [64]. Although we focus on mucosal delivery of neutralizing antibodies, previous reviews have discussed systemic applications of therapeutic antibodies, such as intravenous immunoglobulin (IVIG) delivery [81,82].

Initial studies of passive mucosal vaccination against *C. difficile* toxins yielded promising results in animal models. A 1991 study demonstrated that intragastric vaccination of hamsters with bovine anti-TcdB and anti-TcdA polyclonal IgG antibodies provided complete protection from death [31]. Building off of this work, two subsequent studies would confirm similar protective effects for another preparation of bovine anti-TcdA/anti-TcdB antibodies [79], and they also demonstrate the ability of bovine antibodies to neutralize the toxins in vitro [77]. Besides cow-derived antibodies, avian anti-TcdB and anti-TcdA antibodies were also shown to offer complete protection from death in a hamster model [78]. Between all of these studies, only one [31] reported a problem with CDI relapse post-treatment, suggesting that relapse is not likely to be an issue with passive vaccination.

One of the disadvantages of passive mucosal vaccination, however, is that orally delivered antibodies can be degraded in the GI tract before they reach the site of infection [83,84]. High levels of antibody degradation could reduce the effectiveness of passive vaccination therapies. In the aforementioned 1991 passive vaccination study, for example, anti-TcdA and anti-TcdB IgG stabilized in infant feeding formula offered 100% protection from death, whereas administering antibodies alone offered only 78% protection [31]. Subsequent research examined whether orally delivered anti-TcdA and anti-TcdB antibodies were degraded in the gut. Bovine anti-TcdA and anti-TcdB antibodies were found to be degraded in the GI tract primarily by acid–pepsin digestion, and neither antacids nor proton pump inhibitors improved recovery [85,86]. Despite the harsh gut environment, antibodies recovered from the ileum or stool retain toxin-neutralizing capabilities, indicating that antibodies that resisted digestion could still be therapeutic [85,86]. Since protecting the antibodies in an enteric capsule significantly increases recovery, antibody digestion appears manageable through the development of delayed-release delivery methods [85,86].

#### 4.1.1. Human Trials

Two human trials of passive anti-TcdA and anti-TcdB vaccines have been conducted to date, suggesting this vaccination strategy may be able to prevent recurrent CDI (rCDI). A human trial of the WPC-40 bovine antibody preparation provided full protection from CDI relapse for sixteen patients throughout the 333-day monitoring period with no major adverse effects [79]. A later trial of a similar vaccine design was only moderately successful, however. Experiments with a bovine anti-*C. difficile* IgG cocktail in whey protein (CDIW) found that the vaccine was only equally as effective as metronidazole at preventing rCDI [80]. The study had a similar sample size to the WPC-40 trial (eighteen subjects) and a similar treatment period (two weeks), but only 56% of subjects had responded by seventy days post-treatment.

One possible advantage of WPC-40 over CDIW is that WPC-40 contains a significant amount of secretory IgA (SIgA), which is known to play critical roles in mucosal immune responses [87]. Moreover, the authors of the WPC-40 study noted that SIgA is more resistant to degradation than IgG in the intestinal tract, and the SIgA in WPC-40 likely targets both cellular components and toxins [79,88]. There also appears to be a critical difference in the preparation of WPC-40 versus CDIW. The WPC-40 trial inoculated cows with both formaldehyde-inactivated whole *C. difficile* cells and a toxoid mixture, whereas the CDIW study only inoculated cows with inactivated *C. difficile* cells. Despite the authors’ hypotheses that CDIW might neutralize toxins, the poorer performance of CDIW relative to WPC-40 could have been due to lower toxin-neutralizing capabilities. Future studies into WPC-40, CDIW, or novel passive vaccines should thoroughly characterize the mechanism of protection in vitro to better understand their performance in human trials.

#### 4.1.2. Future Directions for Passive Mucosal Vaccination

Although the above studies show that animal-derived polyclonal antibodies could be harnessed for a passive mucosal vaccine against *C. difficile* toxins, future studies could consider alternative strategies to overcome a number of limitations. As previously mentioned, vaccine antibodies must be protected from degradation in the GI tract. Fortunately, there are many existing technologies for encapsulation that could be evaluated in CDI models [89]. Secondly, animal-derived antibodies raise ethical and cost-related issues for large-scale vaccine production. 

Additionally, the frequent inclusion of milk proteins (due to the use of cows to generate the antibodies in most studies) could present allergy restrictions. Monoclonal antibodies (mAbs) would seem ideal for tackling these problems, as manufacturing is animal-free and would not contain milk allergens. While anti-TcdB mAb (bezlotoxumab) has been approved for clinical use to prevent rCDI [90], both bezlotoxumab and an anti-TcdA mAb (actoxumab) have only been tested as intravenous, systemic passive vaccine candidates. Future studies should be considered for evaluating these mAbs as mucosal vaccines, with extra attention placed on whether bezlotoxumab alone could prevent rCDI. Previous clinical trials showed that intravenous administration of actoxumab and bezlotoxumab did not improve protection from rCDI relative to bezlotoxumab alone [91,92], so perhaps a mucosal vaccine against only TcdB could still be effective.

Besides mAbs, other passive vaccination technologies could be explored. *Lactobacilli*, which naturally inhabit the human microbiome, can be safely consumed, resist degradation in the GI tract, and can be engineered to deliver drug payloads. Recently, *Lactobacillus paracasei* strains were developed that produced anti-TcdB variable domain heavy chain-only (VHH) antibodies either on the cell surface or as a surface-immobilized fragment [93]. While orally delivered VHH alone offered no protection from *C. difficile* infection (likely due to degradation), oral administration of *Lactobacilli* bearing surface-immobilized VHH fragments greatly improved hamster survival (50%) relative to negative controls (0%). Interestingly, the authors chose not to test VHH-secreting strains because the oral administration of VHH fragments failed at doses greater than what the engineered strains would produce in the gut. Future studies would be warranted to test if VHH-secreting strains could be effective, perhaps using more stable VHH fragments. It would also be interesting to examine how long these engineered bacteria persist in the gut, as this raises the possibility of establishing long-lasting protection against rCDI.

### 4.2. Active Mucosal Vaccination against C. difficile Toxins

In contrast to providing a patient with anti-TcdA and anti-TcdB antibodies through passive vaccination, active vaccination stimulates the host immune system to produce antibodies in response to attenuated toxins (toxoids) or toxin fragments presented by the vaccine. Most efforts towards an active vaccine against *C. difficile* toxins have been directed at intramuscularly-delivered toxoids, which previous reviews have examined [94,95]. To summarize, the Pfizer PF-06425090 toxoid vaccine [16,20] and the Valneva PF-06425090 recombinant attenuated toxin vaccine [17] have been found to generate strong antibody responses and provide significant protection from disease, although clinical testing is ongoing. Meanwhile, testing of a Sanofi vaccine (formalin-inactivated TcdA and TcdB) was discontinued [18] due to poor protection [26].

To our knowledge, very few studies have attempted to administer toxoid or recombinant toxin fragments directly to mucosal surfaces. One study administered formalin-inactivated TcdA and TcdB to hamsters through a variety of mucosal and non-mucosal routes [36]. The authors found that rectal vaccination generated low antibody responses and afforded poor protection against death and diarrhea. Subcutaneous, intraperitoneal, and intranasal vaccinations, however, offered full protection from death and limited protection from diarrhea. Ultimately, combining intraperitoneal and intranasal vaccination was necessary to achieve complete protection against both death and diarrhea [36].

As an alternative to administering toxoids at mucosal surfaces, multiple studies have used various bacterial vectors as toxoid delivery systems. In a 1997 publication on CDI vaccination using non-*C. difficile* vectors, Ryan and colleagues orogastrically vaccinated rabbits with an engineered *Vibrio cholerae* strain that expressed a TcdA fragment [96]. The engineered *V. cholerae* successfully colonized the rabbits’ guts, triggering a significant anti-TcdA IgG response. Vaccination offered some protection from TcdA damage in an ileal loop challenge assay, but increasing the toxin levels overwhelmed the limited protective effect of the vaccine [96]. A few years after this study, another group showed that an engineered *Salmonella typhimurium* strain, expressing the C-terminal of TcdA, triggered significant IgG and IgA responses in mice upon intranasal or intragastric vaccination, with intranasal delivery outperforming intragastric delivery [97]. Later, a 2019 study by Winter et al. demonstrated the use of an attenuated, modified *S. typhimurium* strain capable of expressing the receptor binding domains (RBDs) of TcdA and TcdB [40]. The authors found that a combination of intramuscular and oral vaccine doses separated by one week offered full protection from death in a mouse model, while simultaneous intramuscular and oral doses offered 82% protection. In addition, the vaccine stimulated significant IgG levels that inversely correlated with CDI severity, significant IgA responses, and prevented CDI relapse over the three-week post-vaccination monitoring period [40].

In a 2015 study, a *Lactococcus lactis* strain was engineered to express recombinant fragments of TcdA and TcdB [38]. Oral vaccination of mice with the engineered strain reduced mortality and disease severity. Higher titers of anti-TcdA/TcdB antibodies were observed relative to controls, and these antibodies neutralized toxins in vitro. However, subcutaneous injections of the recombinant TcdA and TcdB in mice offered 86% protection compared to 75% in the mucosal vaccine. Since the added complexity of an engineered bacterial delivery system did not outperform the simpler recombinant toxoid vaccine, further refinements of bacterial delivery would be warranted.

Nonetheless, the 75% protection offered by *L. lactis* mucosal vaccine shows great promise, and this can also be said for two more recent studies on bacterially-delivered TcdA/TcdB vaccines. In the first of these studies, Hong and colleagues modified *Bacillus subtilis* spores to express the TcdA C-terminal [98,99]. A combination of oral and sublingual vaccine doses in hamsters prevented *C. difficile* colonization, while an intramuscular toxoid vaccine did not [98]. Oral vaccination of mice with the modified spores generated a robust IgA and IgG response against TcdA, as well as cross-reactivity with TcdB [99]. The vaccine protected 75% of hamsters from death, with all surviving hamsters being immune from re-challenge with *C. difficile* [99]. Not only did these studies demonstrate the effectiveness of a *B. subtilis* vaccine delivery platform, but they also showed that a TcdA antigen could induce antibody responses capable of neutralizing both TcdA and TcdB [98,99]. This approach could greatly simplify future toxin vaccines for CDI.

Regardless of whether toxin-based vaccines are administered via mucosal routes or through more traditional methods, there are a number of challenges that could hamper their ultimate effectiveness. Both animal models [34] and clinical trials [100] have demonstrated that toxin-based vaccines do not prevent colonization. Colonization can occur even in the presence of high systemic anti-toxin IgG levels, suggesting that anti-toxin vaccines may unintentionally promote asymptomatic carriage [101,102]. Asymptomatic carriage has not been closely studied in mucosal toxin vaccine models, but prior observations with toxin vaccines would seem to indicate that this weakness would remain.

Additionally, the long-run effectiveness of toxoid vaccines is uncertain. Anti-TcdB antibodies from recovered patients displayed moderate affinity and limited neutralization capabilities [103], so it is unclear whether anti-toxin vaccines will protect from later exposures. Current designs may be less effective against hypervirulent strains, which express the binary toxin (CDT) and cause severe disease [104]. An intramuscular vaccine made from attenuated TcdA, TcdB, and CDT protected hamsters from a hypervirulent strain, whereas vaccinating without CDT was ineffective [105]. Most vaccines remain untested against hypervirulent strains, but these findings suggest that future vaccines should consider including binary toxin components to effectively prevent severe infections. In summary, mucosal vaccines against *C. difficile* toxins alone may not provide comprehensive protection against diverse strains, colonization, and multiple exposures. However, vaccinating against toxins and *C. difficile* surface components may provide the benefits of toxin neutralization along with greater colonization protection.

## 5. Surface-Antigen Mucosal Vaccine

**Figure 3:**
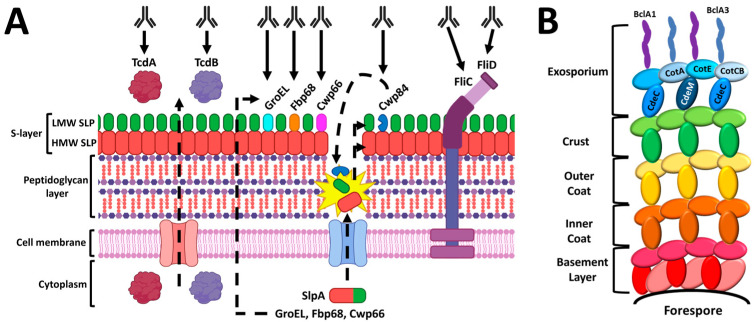
*C. difficile* vaccine candidates. Diagram of toxin and cell-surface antigens (**A**) and spore coat proteins (**B**) currently under investigation. The structure of the *C. difficile* cell envelope shown here (**A**) is based, in part, on the illustrations provided by a prior review [109]. Dotted lines indicate generalized transport mechanisms that are not completely understood. The S-layer is composed of the high molecular weight S-layer protein (HMW SLP) and an outer layer of low molecular weight SLPs (LMW SLPs) and additional proteins [110]. The CD0873 lipoprotein, despite being a mucosal vaccine candidate, is not shown because its precise position in the S-layer of *C. difficile* has not yet been determined [109]. All spore-coat vaccine candidates (**B**) are localized in the exosporium layer. The spore structure diagram (**B**) was based on a recent review by Paredes-Sabja et al. [111]. A publication license was obtained for BioRender content used in Figure 3A on 2 January 2023 (agreement number LX24UCVM8E).

CDI patients generate IgG responses to cell–surface antigens [106], presenting the opportunity to develop vaccines against vegetative cells and/or spores. One method of targeting surface components involves vaccination with *C. difficile* membrane preparations. For example, rectal vaccination with a membrane preparation containing the adhesin Cwp66 and S-layer proteins reduced *C. difficile* colonization in mice [39]. Another group developed a nontoxigenic *C. difficile* membrane fraction (ntCDMF) as a rectal mucosal vaccine that was also protective in a mouse model [107]. While ntCDMF contains SleB, a common *C. difficile* membrane protein, it is unclear what specific antigens were protective in the study [107,108]. Other studies have identified more specific surface-antigen candidates for vaccine development, such as S-layer-localized proteins, flagellar components, and spore coat proteins. We summarize these targets and their approximate location on the *C. difficile* bacterium or spore in Figure 3.

### 5.1. Surface-Layer Proteins

Surface-layer (S-layer) proteins (SLPs) cover the outside of the *C. difficile* bacterium, and they are implicated in bacterial adhesion and immune activation [109]. The S-layer is primarily comprised of high molecular weight SLP (HMW SLP) and low molecular weight SLP (LMW SLP), which are formed by the cleavage of SlpA by the protease Cwp84 [109,112]. Vaccination with these antigens has been challenging thus far. Intraperitoneal vaccination with HMW SLP and LMW SLP in mice and hamsters was not strongly immunogenic or protective, even using Ribi or cholera toxin adjuvants [35]. Due to this result, the authors noted the need for adjuvant alternatives in future studies. Intra-rectal vaccination of mice and hamsters with SlpA and cholera toxin, on the other hand, significantly reduced colonization in mice, although protection from death was not significant in a hamster model [113]. Both studies proposed that alternative adjuvants could improve the performance of SLP vaccination strategies [35,113]. Since orogastric passive vaccination of hamsters with anti-SLP antibodies has been found to prolong survival [33], finding ways to generate a stronger antibody response could make active SLP vaccination viable.

*C. difficile* encodes a number of SlpA paralogues classified as cell wall proteins (CWPs) [109], which contain a specific cell wall binding repeat motif [114]. The cell wall protein Cwp84, which is involved with S-layer processing [112], is immunogenic in CDI patients [115], and rectal vaccination with Cwp84 reduced colonization in mice [39]. A follow-up study with hamsters found that rectal vaccination protected animals from death, but most animals were still colonized by *C. difficile* [32]. While rectal vaccination showed some promise, oral delivery of Cwp84 would be preferable in a clinical setting due to greater simplicity. At first, intragastric delivery of a Cwp84 vaccine failed to offer any protection from CDI, likely due to degradation in the GI tract [39]. A later study encapsulated Cwp84 with pectin beads, which greatly improved hamster survival (40%) relative to both unencapsulated Cwp84 vaccination and unvaccinated controls (both 0%) [116]. Although oral vaccination offered roughly the same level of protection as rectal vaccination (33–50%), anti-Cwp84 antibody levels did not correlate with survival [32]. Moreover, the ability of *C. difficile* to colonize animals despite mucosal vaccination with Cwp84 [39] suggests that it cannot be used as a standalone vaccine candidate. Future studies could consider the suitability of alternative cell wall proteins, such as Cwp66. Although Cwp66 has yet to be tested as a mucosal vaccine, it appears promising due to its surface-exposed [117] C-terminal region that is more immunogenic than both TcdA and TcdB [106].

Another more promising mucosal vaccine candidate is CD0873. This surface-localized lipoprotein is immunogenic and is thought to support *C. difficile* colonization [118,119,120]. Orally administered encapsulated CD0873 was found to stimulate significant SIgA and IgG levels in hamsters, and the vaccine-induced antibody responses blocked *C. difficile* adherence [121]. This strong immune response to CD0873 offered 80% protection from death in the study [121]. A subsequent study by Karyal et al. demonstrated the use of liposomes to more effectively deliver the CD0873 antigen [122]. Not only does this appear to be the first use of a liposome delivery system for CDI vaccination, but this delivery strategy produced a more effective neutralizing antibody response as measured by greater inhibition of *C. difficile* adherence by vaccine-induced antibodies [122]. The authors indicated that the increased effectiveness of liposome-displayed CD0873 could be due to reduced protein aggregation, greater uptake of the vaccine by M cells, and interactions with macrophages due to the specific liposome composition. Future studies into CD0873 vaccines would be warranted to see how the increased antibody neutralization response impacts survival and colonization in animal vaccination models. However, the evidence so far suggests that liposome-based delivery could be a promising method for increasing the effectiveness of mucosal vaccines against *C. difficile* surface components.

A few other surface protein candidates could be considered for further mucosal vaccine development. GroEL is a heat-shock protein that can be secreted or surface-expressed, and it is suspected to be involved with *C. difficile* colonization [123]. Intranasal vaccination against GroEL was immunogenic and reduced colonization in mice [124]. Intrarectal vaccination of hamsters prolonged survival, but it did not offer strong protection [124]. Lastly, the fibronectin-binding protein Fbp68, which is likely involved in *C. difficile* adhesion, is an understudied vaccine candidate [106]. Most CDI patient sera contain anti-Fbp68 antibodies, and the protein was found to be more immunogenic than TcdA and TcdB [106]. Future studies could address the suitability of Fbp68 as a mucosal vaccine antigen.

### 5.2. Flagellar Proteins

The flagellin FliC and flagellar cap FliD are involved with bacterial attachment [125]. Compared to healthy individuals, CDI patients exhibit significantly higher levels of anti-FliC and anti-FliD antibodies [106]. Total anti-FliD antibodies were comparable to anti-TcdA and anti-TcdB antibodies, although anti-FliC antibody levels were the lowest for the seven antigens examined [106]. A later study confirmed that anti-FliD and anti-FliC antibodies could be detected at least two weeks post diagnosis, suggesting that these proteins are actively utilized by *C. difficile* during infection [115].

So far, only a few studies have tested FliC and FliD mucosal vaccines. Rectal administration of FliD generated the highest level of IgA and only slightly lower IgG levels compared with parenteral delivery in mice [39]. Mice that were intrarectally vaccinated with either a flagellar preparation (FliC, FliD, and other flagellar components) or a combination of Cwp84 and FliD showed reduced intestinal colonization [39]. Further animal studies to address how these vaccination strategies might reduce disease mortality and recurrence would be warranted to better understand the capabilities of FliC or FliD vaccines. For example, intraperitoneal injection of recombinant *C. difficile* FliC in mice was immunogenic and offered complete protection from CDI [126], but mucosal administration has not been tested.

It should be noted that FliC/FliD vaccine performance may vary widely depending on the delivery route. Despite the aforementioned successes with intrarectal vaccination, intranasal and intragastric FliD vaccination was not strongly immunogenic (even when the latter method was combined with antigen encapsulation) [39]. Further investigation into flagellar protein mucosal vaccines could use novel delivery methods to improve performance. For example, one study generated fusion proteins of *C. difficile* FliD with the *B. subtilis* spore coat proteins CotB, CotC, CotG, and CotZ [127]. These fusion proteins can be expressed on *B. subtilis* spores, paving the way for potential oral spore-based vaccines against FliD.

### 5.3. Spore Coat Proteins

Numerous proteins are responsible for the spore coat structure, such as CotA, CotE, and CotCB, among others [128] Several spore proteins can be localized at the spore surface with antibodies [128], and sera from goats injected with *C. difficile* spores were shown to be reactive against strain R20291 spore components [129,130]. Although spore proteins are difficult to express due to glycosylation [131], developing anti-spore vaccines could offer a powerful tool to prevent *C. difficile* colonization.

Two potential spore antigens for mucosal vaccination include CdeC and CdeM [132]. Both are abundantly found in the exosporium and are unique to *C. difficile* [131,133]. One study found that CdeC or CeM were immunogenic in mice, and intraperitoneal vaccination offered strong protection in both mice and hamsters [131]. Moreover, both vaccines significantly reduced spore shedding in mice [131]. Future studies of CdeC and CdeM as mucosal vaccines would be warranted to determine if mucosal administration could offer further performance improvements.

Recent studies demonstrated, perhaps unintentionally, the promise of mucosal vaccines targeting spore proteins, such as CdeC. We previously discussed a study demonstrating the use of modified *B. subtilis* spores expressing the TcdA C-terminal [98,99]. After demonstrating the effectiveness of the vaccine in vivo, a follow-up study by the same group determined that antibodies generated against the recombinant TcdA fragment in their vaccine were cross-reactive to cell-surface components and spore proteins of *C. difficile* (specifically the spore protein CdeC and the dehydrogenases AdhE1 and LdhA) [98]. Vaccine-generated antibodies were found to inhibit *C. difficile* adherence to intestinal cells [98]. This work suggests that mucosal vaccines targeting toxins, surface components, and spore proteins simultaneously could be highly effective for rCDI prevention.

Unfortunately, other spore coat targets have proven more difficult to use for vaccine development. One study intranasally delivered the C-terminal domain of the spore surface protein BclA2 (BclA2_CTD_) into mice. A second vaccine design was tested by adsorbing BclA2_CTD_ to *Bacillus subtilis* (*B. subtilis*) spores [134]. Both free and adsorbed BclA2_CTD_ triggered similar and significant IgG responses in mice after two immunizations, but neither vaccine blocked colonization or mitigated CDI symptoms in vivo.

Similar strategies were applied to a recombinant BclA3 vaccine with only slightly better performance [135]. Intranasal delivery of the C-terminal domain of the spore surface protein BclA3 (BclA3_CTD_) produced IgG responses in mice. This time, however, a *B. subtilis* spore-displayed BclA3_CTD_ was less immunogenic than the protein alone. Administration of the free BclA3_CTD_ vaccine reduced spore levels in murine feces slightly faster than controls and prevented weight loss, a noticeable improvement on the BclA2_CTD_ study. However, the BclA3_CTD_ vaccine was unable to reduce diarrhea prevalence, diarrhea severity, spore load in the gut, or toxin levels in feces. Another group observed similar results—that their intranasal, recombinant BclA3 vaccine could not mitigate symptoms, prevent colonization, or reduce the spore load [136]. Expressing the BclA3 fragment on *B. subtilis* spores did not improve results [136]. Since the glycosylation of recombinant BclA3 is not representative of the dominant glycan structure on the spore coat [136], this may contribute to the poor performance.

Other antigens that have proven challenging so far include the exosporium protein BclA1 [130], the spore cortex enzyme SleC (involved with germination) [137], and the spore coat protein CotA [128]. These antigens have demonstrated mixed results using intraperitoneal vaccination, while mucosal administration has yet to be tested. For example, vaccination with CotA offered partial (60%) protection in mice, but spore shedding did not change significantly. Meanwhile, intraperitoneal BclA1 or SleC vaccination was not protective in mice, although SleC vaccination did reduce spore shedding [131]. The authors cited several reasons for the shortcomings of these antigens. BclA1 was likely ineffective against the RT027 *C. difficile* used in the test because RT027 strains express truncated BclA1 [131]. Meanwhile, CotA is not expressed on all spores [131]. Lastly, the authors noted a study reporting that SleC-mutant spores could still germinate, potentially offering a pathway for *C. difficile* to bypass the effects of SleC vaccination [138]. Taken together, the above studies suggest that developing an effective vaccine using spore coat proteins presents many challenges, as the current array of antigens may not provide the protection required of a *C. difficile* mucosal vaccine. However, future research should consider the intranasal delivery methods presented in the BclA2 and BclA3 vaccination studies. Even though intranasal vaccination with spore coat proteins is not strongly protective based on current findings, additional refinement or the use of other antigens could yield an effective mucosal vaccine that could be easily administered in a clinical setting.

## 6. Whole-*C. difficile* Mucosal Vaccine

Recently, our research group was the first to report an engineered non-toxigenic *C. difficile* (NTCD) strain as a mucosal vaccine against CDI [30]. NTCD has previously been explored as a CDI prevention measure, as NTCD may compete with toxigenic strains for the same niche in the gut [139]. In both hamster and mouse models, colonization with an NTCD strain provided protection from challenge with toxigenic *C. difficile* [140]. A clinical trial using the NTCD strain M3 demonstrated that NTCD was safe, could colonize the human gut, and provided protection from CDI [141]. In our study, the NTCD strain CCUG37785 was modified to express a recombinant protein mTcd138 composed of the glucosyltransferase domain (GTD) and cysteine proteinase domain (CPD) of TcdB, as well as the receptor binding domain (RBD) of TcdA. We found that oral administration of spores from the modified strain (NTCD_mTcd138) ellicited a significant antibody response against toxins and surface components, such as FliC and FliD. Moreover, NTCD_mTcd138 vaccination offered full protection from CDI in mice and >60% protection in hamsters [30]. 

In a subsequent publication, our group again proposed a new design for a modified NTCD vaccine expressing toxin subunits [142]. For this design, CCUG37785 was altered to express the RBD, the GTD domain, and the CPD of TcdB, as well as the RBD of TcdA (known altogether as the NTCD_Tcd169 vaccine). In addition to generating antibody responses against both toxins, FliC, and FliD, NTCD_Tcd169 also elicited anti-SlpA and anti-Cwp2 antibodies, demonstrating a broad neutralization capability. Similarly to the previous vaccine, NTCD_Tcd169 offered complete protection from CDI and reduced fecal spore load in mice.

Building off of our work on NTCD-based vaccines, another group sought to develop a modified NTCD vaccine that placed special emphasis on neutralizing colonization factors to block early pathogenesis [143]. The NTCD strain T7 was modified to express the colonization factor CD0873 and a domain of TcdB. The researchers cited prior studies demonstrating that an orally delivered recombinant protein vaccine of CD0873 could induce an antibody response to block colonization [121,122]. Likewise, a recombinant protein vaccine using the TcdB RBD offered full protection in an earlier study [37]. Oral administration of modified T7 spores in hamsters triggered a strong systemic and intestinal immune response, as measured by IgG and IgA levels [143]. Antibodies recovered from intestinal fluid and sera significantly reduced the adhesion of toxigenic *C. difficile* to Caco-2 cells [143]. Since our research group also demonstrated that oral vaccination of hamsters with an unmodified NTCD strain (CCUG37785) also offered near-complete protection from CDI while also reducing spore shedding [144], this raises the opportunity for future studies to compare the effectiveness of NTCD vaccines that express recombinant antigens versus those that do not. Regardless of whether modified or unmodified strains of NTCD are considered moving forward, it will be important to determine beforehand the risk of toxigenic conversion of the NTCD strains, as well as how long these strains will persist in patients following vaccination [145,146].

## 7. Mucosal Vaccination of Vulnerable Patient Populations

The incidence of primary and recurrent CDI is generally higher in immunocompromised populations (e.g., organ transplants, cancer patients) [147]. Moreover, patients suffering from inflammatory bowel disease (IBD) are also at an increased risk of various gastrointestinal infections [148]. Such vulnerable patients stand to benefit significantly from a CDI vaccine, but at the same time, vaccine effectiveness and safety in these populations are major concerns. Due to the lack of data on *C. difficile* vaccination and IBD, there are no specific guidelines for how to apply preventative therapies against CDI specifically [149]. More generally, vaccination recommendations for IBD patients suggest that, so long as a given patient is not immunosuppressed, live or non-live vaccines are generally safe [150]. However, future research should evaluate the efficacy and safety profile of *C. difficile* vaccines in IBD patients specifically. 

Regarding immunocompromised patients, a systematic review of vaccine responses in diverse immunocompromised populations found that the effectiveness of vaccines varied by the exact nature of the immune deficiency. For example, patients with solid tumors or immune-mediated inflammatory diseases responded similarly to vaccines when compared with non-immunocompromised patients, whereas B-cell deficiencies were associated with poor responses [151]. Regarding safety concerns, live-attenuated vaccines against viral (e.g., polio and varicella [152]) and bacterial (e.g, BCG [153]) may present a risk of vaccine-induced disease in immunocompromised patients. Non-live and component vaccines are generally considered to be safe for most patients [152,154]. Unfortunately, there is a need for data on the safety and effectiveness of *C. difficile* vaccines in immunocompromised patients [155], and we are not aware of any studies that have directly addressed these questions. If trends for other vaccines apply to *C. difficile* vaccines, then component vaccine technology would likely be easiest to use in both immunocompetent and immunocompromised patients. Fortunately, immunocompromised patients could rely on other treatments until a safe, effective vaccine is validated in vulnerable patient subgroups. For example, a recent systematic review of fecal microbiota transfer (FMT) found that 87% of immunocompromised patients recovered after only one round of FMT, with 93% of immunocompromised patients recovering after several rounds of therapy [156]. The frequency of major adverse effects was no greater than in immunocompetent patients treated with FMT [156].

## 8. Concluding Remarks

Due to the growing threat of *C. difficile*, effective vaccines are needed to prevent outbreaks. Several *C. difficile* antigens have been evaluated, thus far, as potential vaccine candidates, including toxins, surface proteins, spore proteins, and engineered *C. difficile*. Although current data suggest certain strengths and weaknesses in relation to these antigens (summarized in Table 1), much more research is needed to fully evaluate the performance of individual vaccine antigens, as well as combined vaccine regimens for optimal protection from both colonization and severe disease. A significant portion of mucosal vaccine research for CDI has been conducted using in vitro methods and animal models, while comparatively few mucosal vaccines have been applied in humans to prevent CDI. 

While considerable groundwork has already been performed testing both passive and active vaccination against *C. difficile* toxins (both through mucosal and non-mucosal delivery), it does not appear that toxin-based vaccines can prevent colonization. Future designs need to address this weakness to halt the spread of CDI in healthcare facilities through asymptomatic carriage. Combining toxin antigens with surface antigens of vegetative cells and/or spores may be the most promising strategy to facilitate the clearance of *C. difficile*. The effectiveness of surface–antigen vaccine candidates against hypervirulent strains should also be given increased attention to determine if binary toxin antigens should be included in a CDI vaccine.

Regarding how to deliver a mucosal vaccine against *C. difficile*, each method available has a number of advantages and downsides. While many animal models successfully made use of intrarectal delivery, patient compliance could be an issue in a clinical setting. Intranasal vaccination would be far easier to administer, but more work is needed to determine which *C. difficile* antigens will be most protective using this strategy. Oral delivery of antigens would also be simpler than intrarectal delivery, and multiple oral vaccines have shown promise in testing so far. Perhaps the greatest challenge of oral vaccination is preventing the degradation of antigens in the GI tract. Fortunately, either encapsulation or delivery by orally administered recombinant bacteria appears to mitigate the problem of degradation. Recombinant bacteria, especially non-toxigenic *C. difficile*, could combine the advantages of stimulating strong mucosal immunity with the added bonus of occupying the metabolic niche that toxigenic *C. difficile* would attempt to use upon colonization of the gut. Moreover, engineered NTCD strains can present multiple antigens simultaneously, simplifying the challenge of producing and delivering multiple recombinant proteins to the mucosa. Future efforts could explore the mechanisms of protection offered by engineered bacteria, as well as alternative delivery methods for expressing *C. difficile* antigens at mucosal surfaces.

## Figures and Tables

**Table 1:** Advantages and disadvantages of potential *C. difficile* mucosal vaccine candidates.

Target Antigen	Advantages	Disadvantages
TcdA and TcdB	Direct presentation of antigens at mucosal sites by engineered commensal bacteria [38,40,97] TcdA/TcdB fragment-expressing commensals provide strong protection from death in animal models [38,40,97] TcdA C-terminal-expressing *C. difficile* spores provided colonization resistance and cross-reactivity to TcdB [98,99]	Rectally administered, formalin-deactivated TcdA and TcdB generated poor antibody responses and poor protection [36] No protection from colonization [34,100] May promote asymptomatic carriage of CDI [101,102] Long-term binding effectiveness of vaccine induced anti-TcdB antibodies is unclear [103]. May not protect against hypervirulent *C. difficile* [105]
*C. difficile* membrane preparation	Rectal vaccination of mice with a *C. difficile* membrane fraction reduced colonization [39] Intrarectal vaccination of mice with ntCDMF reduced fecal bacterial load and decreased death [107,108]	Which antigens were protective in these studies has not been fully determined [39,107,108]
SlpA	Intra-rectal vaccination of mice reduced colonization [113]	Immunogenicity varies widely based on adjuvant [35,113] Protection from death was not significant in hamsters [113]
Cwp84	Immunogenic in CDI patients [115] Rectal vaccination reduced colonization in mice [39] Rectal vaccination reduced hamster deaths [39] Encapsulated Cwp84 is stable in the GI tract [39]	Rectally vaccinated hamsters are still colonized [39] Susceptible to degradation in the gut [39] Anti-Cwp84 antibody levels did not correlate with survival in a hamster vaccine model [32]
Cwp66	Cwp66 C-terminal region is surface-exposed [117] and is more immunogenic than TcdA or TcdB [106]	Cwp66 has yet to be tested as a mucosal vaccine
CD0873	Encapsulated CD0873 stimulated strong SIgA and IgG responses in hamsters [121] Anti-CD0873 antibodies blocked *C. difficile* adherence and protected from death [121] Liposome delivery of CD0873 produced even greater antibody responses than encapsulated antigen [122]	The liposome delivery mechanism needs further characterization [122]
GroEL	Intranasal vaccination reduced colonization in mice [124]	Intrarectal vaccination of hamsters prolonged survival, but it did not offer strong protection [124]
Fbp68	Immunogenic in CDI patients [106] More immunogenic than TcdA or TcdB [106]	Untested as mucosal vaccine
FliC and FliD	CDI patients produce strong antibody responses to FliC and FliD [106] Rectal administration of FliD generated significant IgA and IgG levels in mice [39] May be able to reduce colonization if used with other surface antigens [39]	Intraperitoneal FliC injections were protective in mice [126], but mucosal administration has not been tested Intranasal and intragastric FliD vaccination was not strongly immunogenic [39] While *B. subtilis* spores expressing FliD fragments have been developed, their effectiveness has not been evaluated [127]
CdeC and CdeM	Abundant in exosporium and unique to *C. difficile* [131,133] Both proteins are immunogenic in mice [131] Intraperitoneal vaccination offered strong protection in both mice and hamsters [131] Intraperitoneal vaccination with either protein reduced spore shedding in mice [131] Other vaccine designs related to B. subtilis spores, expressing the TcdA C-terminal [98,99], generated antibody responses against CdeC	Not tested as mucosal vaccine Other vaccine designs, such as *B. subtilis* spores expressing the TcdA C-terminal [98,99], generated anti-CdeC antibodies without intentionally including CdeC as an antigen
BclA2	An intranasally delivered BclA2 fragment (BclA2_CTD_) protected mice from death [134] Free and spore-adsorbed BclA2_CTD_ triggered significant murine IgG responses [134]	Neither free nor spore-adsorbed BclA2_CTD_ blocked colonization or mitigated CDI symptoms in vivo [134]
Bcla3	An intranasally delivered BclA3 fragment (BclA3_CTD_) was immunogenic in mice [135] Intranasal delivery of the C-terminal domain of the spore surface protein BclA3 (BclA3_CTD_) produced IgG responses in mice Vaccination with free BclA3_CTD_ prevented weight loss in mice [135].	Spore-displayed BclA3_CTD_ was less immunogenic than free BclA3_CTD_ and does not appear to improve vaccine performance [135,136] BclA3_CTD_ vaccination in mice was unable to reduce diarrhea prevalence, diarrhea severity, spore load in the gut, or toxin levels in feces [135,136] Recombinant BclA3 glycosylation is not representative of the dominant glycan structure on the spore coat, possibly contributing to poor vaccine performance [136]
Bcla1		Intraperitoneal vaccination was not protective in mice [131] RT027 strains express truncated BclA1 [131], possibly requiring a separate BclA1 vaccine for these strains Not evaluated as a mucosal vaccine
SleC	Intraperitoneal vaccination reduced spore shedding in mice [131]	Intraperitoneal vaccination did not protect mice from death [131] SleC-mutant spores can still germinate [138] Not evaluated as a mucosal vaccine
CotA	Intraperitoneal vaccination with CotA protected mice from death [131]	Intraperitoneal CotA vaccination did not reduce spore shedding [128] CotA is not expressed on all spores [131] Not evaluated as a mucosal vaccine
Non-toxigenic *C. difficile* (NTCD)	NTCD may compete with toxigenic strains for the same niche in the gut [139] NTCD can be modified to express fragments of C. difficile toxins and colonization factors [30,142,143] Oral vaccination with modified NTCD strains ellicited antibody responses against toxins, surface components, and colonization factors [30,142,143] Vaccination with modified NTCD strains offers strong, sometimes complete, protection from death in mice in hamsters [30,142]	Concerns over toxigenic conversion of administered NTCD strains [145] Long-term protection may wane if NTCD is not retained after completing therapy [146]

## Data Availability

Not applicable.

## Supplementary Materials

The following supporting information can be downloaded at: https://www.mdpi.com/article/10.3390/vaccines11050887/s1, Table S1: *C. difficile* vaccine candidates in clinical trials. References [16,17,19,20,21,22,23,24,25,26,27] are cited in Supplementary Materials.
