# Supplementary material for: Mucosal Vaccination Strategies against Clostridioides difficile Infection
Source: Vaccines (Basel). 2023 Apr 23;11(5):887. doi: 10.3390/vaccines11050887 (PMC10223210; doi:10.3390/vaccines11050887)
Supplement: Supplementary file 1 [file vaccines-11-00887-s001.zip › vaccines-2223937-supplementary.pdf]

vaccines

MDPI

Supplementary Information

Mucosal Vaccination Strategies Against *Clostridioides difficile* Infection

Joshua Heuler , Harish Chandra[2] and Xingmin Sun *

Supplementary Table S1 . *C. difficile* vaccine candidates in clinical trials.

Name (Manufacturer)	Strategy	Status	Findings	References
PF-06425090 (Pfizer)	Toxoid; genetically and chemically detoxified TcdA and TcdB	Phase III	*In vitro*	
			• Produced mutant TcdA and TcdB using an expression vector in a non-sporulating *C. difficile* strain that does not carry either *tcdA* or *tcdB*<br>• Mutant TcdA (D285A/D287A) and TcdB (D286A/D288A) lacked glucosyltransferase and autoproteolytic processing activities<br>• Toxin mutants were found to have greatly reduced toxicity to IMR-90 fibroblasts<br>• Formalin inactivation removed residual toxicity of mutant TcdA and TcdB	NCT01706367 [1]
			In vivo	
			• Inactivated, mutant TcdB and TcdA were immunogenic in hamsters<br>• Vaccine offered 60% protection from death compared to 0% in unvaccinated hamsters	NCT01706367 [1]
			Human trials	
			• In 50-85 year olds, vaccine was immunogenic and safe<br>• Vaccine was less immunogenic with aluminum hydroxide adjuvant<br>• No dose response observed	NCT01706367 [2]
			• Vaccine was safe and immunogenic in 65–85 year olds<br>• Optimized vaccine dose amount and time interval between the three doses	NCT02561195 [3]
			• Vaccine candidate offered complete protection from severe CDI as well as reduced CDI episode length<br>• Only 31-49% efficacy at preventing primary CDI, which did not meet study goals	NCT03090191
VLA84 (Valneva)	recombinant chimeric TcdB and TcdA binding domains	Phase II	*In vitro*	
			• Vaccine-induced antibodies neutralized toxin activity in T84 cells	[4]
			• Vaccination of mice, hamsters, and monkeys with fusion protein induced antibodies that could neutralize toxins in a Vero cell model<br>• Dose-dependent response observed between antibody neutralization capability and use of vaccine adjuvant, aluminum hydroxide	[5]
			In vivo	
			• Vaccine provided mice with complete protection from death from TcdA challenge<br>• Vaccine protection from death by TcdB challenge in mice was strong<br>• In hamsters, vaccine offered complete protection from non-lethal dose of *C. difficile* even without adjuvant, but adjuvant was required for complete protection from lethal *C. difficile* dose	[5]
			Human trials	
			• VLA84 was safe and immunogenic in adults<br>• IgG antibodies responses lasted at least 6 months	[4]

Continued.

Supplementary Table S1 Cont.

Name (Manufacturer)	Strategy	Status	Findings	
Cdiffense (Sanofi/Pasteur)	Formalin-inactivated TcdA and TcdB	Phase III halted	*In vitro*	
			• Anti-TcdA and anti-TcdB antibodies triggered by the vaccine could neutralize the toxic effect on IMR-90 fibroblasts	[6]
			Human trials	
			• Parenteral administration of TcdA toxoid triggered significant, protective levels of anti-TcdA antibodies in healthy individuals	[7]
			• Vaccine was safe an immunogenic in patients with recurrent CDI<br>• Prevented recurrence of CDI, albeit sample size was small (3 patients)	[6]
			• Candidate was immunogenic and safe in a cohort of healthy individuals<br>• Adjuvant use increased immunogenicity, but the effect was not significant	[8]
			• Vaccine was immunogenic and safe in patients 18-55 years	NCT00772954
			• Vaccine was immunogenic and safe in patients 18-55 years old (NCT001278030 and >65 years old (NCT00214461)	NCT00127803<br>NCT00214461<br>[9]
			• Optimized vaccine dose amount and time interval between the three doses<br>• Determined that vaccine was more immunogenic in people aged 40–75 years with aluminum hydroxide adjuvant	NCT01230957<br>[10]
			• Phase III testing found that the vaccine candidate did not provide protection from CDI	NCT00772343<br>[11]

References

1. Donald, R.G.; Flint, M.; Kalyan, N.; Johnson, E.; Witko, S.E.; Kotash, C.; Zhao, P.; Megati, S.; Yurgelonis, I.; Lee, P.K. A novel approach to generate a recombinant toxoid vaccine against Clostridium difficile. *Microbiology* **2013**, *159*, 1254.
2. Sheldon, E.; Kitchin, N.; Peng, Y.; Eiden, J.; Gruber, W.; Johnson, E.; Jansen, K.U.; Pride, M.W.; Pedneault, L. A phase 1, placebo-controlled, randomized study of the safety, tolerability, and immunogenicity of a Clostridium difficile vaccine administered with or without aluminum hydroxide in healthy adults. *Vaccine* **2016**, *34*, 2082-2091.
3. Kitchin, N.; Remich, S.A.; Peterson, J.; Peng, Y.; Gruber, W.C.; Jansen, K.U.; Pride, M.W.; Anderson, A.S.; Knirsch, C.; Webber, C. A phase 2 study evaluating the safety, tolerability, and immunogenicity of two 3-dose regimens of a Clostridium difficile vaccine in healthy US adults aged 65 to 85 years. *Clinical Infectious Diseases* **2020**, *70*, 1-10.
4. Bézay, N.; Ayad, A.; Dubischar, K.; Firbas, C.; Hochreiter, R.; Kiermayr, S.; Kiss, I.; Pinl, F.; Jilma, B.; Westritschnig, K. Safety, immunogenicity and dose response of VLA84, a new vaccine candidate against Clostridium difficile, in healthy volunteers. *Vaccine* **2016**, *34*, 2585-2592.
5. Tian, J.-H.; Fuhrmann, S.R.; Kluepfel-Stahl, S.; Carman, R.J.; Ellingsworth, L.; Flyer, D.C. A novel fusion protein containing the receptor binding domains of C. difficile toxin A and toxin B elicits protective immunity against lethal toxin and spore challenge in preclinical efficacy models. *Vaccine* **2012**, *30*, 4249-4258.

6. Sougioultzis, S.; Kyne, L.; Drudy, D.; Keates, S.; Maroo, S.; Pothoulakis, C.; Giannasca, P.J.; Lee, C.K.; Warny, M.; Monath, T.P. Clostridium difficile toxoid vaccine in recurrent C. difficile-associated diarrhea. *Gastroenterology* **2005**, *128*, 764-770.
7. Aboudola, S.; Kotloff, K.L.; Kyne, L.; Warny, M.; Kelly, E.C.; Sougioultzis, S.; Giannasca, P.J.; Monath, T.P.; Kelly, C.P. Clostridium difficile vaccine and serum immunoglobulin G antibody response to toxin A. *Infection and immunity* **2003**, *71*, 1608-1610.
8. Kotloff, K.L.; Wasserman, S.S.; Losonsky, G.A.; Thomas Jr, W.; Nichols, R.; Edelman, R.; Bridwell, M.; Monath, T.P. Safety and immunogenicity of increasing doses of a Clostridium difficile toxoid vaccine administered to healthy adults. *Infection and immunity* **2001**, *69*, 988-995.
9. Greenberg, R.N.; Marbury, T.C.; Foglia, G.; Warny, M. Phase I dose finding studies of an adjuvanted Clostridium difficile toxoid vaccine. *Vaccine* **2012**, *30*, 2245-2249.
10. de Bruyn, G.; Saleh, J.; Workman, D.; Pollak, R.; Elinoff, V.; Fraser, N.J.; Lefebvre, G.; Martens, M.; Mills, R.E.; Nathan, R. Defining the optimal formulation and schedule of a candidate toxoid vaccine against Clostridium difficile infection: A randomized Phase 2 clinical trial. *Vaccine* **2016**, *34*, 2170-2178.
11. de Bruyn, G.; Gordon, D.L.; Steiner, T.; Tambyah, P.; Cosgrove, C.; Martens, M.; Bassily, E.; Chan, E.-S.; Patel, D.; Chen, J. Safety, immunogenicity, and efficacy of a Clostridioides difficile toxoid vaccine candidate: a phase 3 multicentre, observer-blind, randomised, controlled trial. *The Lancet Infectious Diseases* **2021**, *21*, 252-262.
